# Tile-Ippokratis: The Experience of an Ehealth Platform for the Provision of Health Care Services in the Island of Chios and Cyprus

**DOI:** 10.1155/2010/357156

**Published:** 2010-09-07

**Authors:** Homer Papadopoulos

**Affiliations:** Department of Applied Technologies, National Center for Scientific Research “Demokritos”, 15310 Attiki, Greece

## Abstract

Tile-Ippokratis proposed an integrated platform for the provision of low-cost ehealth services to citizens in southeast Mediterranean area (Island of Chios and Cyprus). The aim of the paper is to present the architecture, the design, and the evaluation results of this platform. The platform based on already evaluated state-of-the-art mobile ehealth systems and using wireless and terrestrial telecommunication networks is able to provide the following health care services: (i) telecollaboration and teleconsultation services between health care personnel and between health care personnel and patients and (ii) ehealth services for “at risk” citizens such as elderly and patients with chronic diseases (Island of Chios) and postsurgery patients (Cyprus). The ehealth systems supported capabilities for vital signal measurements (ECG 1 lead, SPO2, HR, BP, weight, and temperature), an Electronic Patient Record (EPR) infrastructure, and video conference, along with communication gateways for data transmission over ADSL, GPRS, and WLAN networks.

## 1. Introduction

Ehealth is an umbrella term which refers to the use of information and communication technologies (ICTs) for the provision of health services from distance. Ehealth, distinguished into eCare, eLearning, eSurveillance, and eGoverment/eAdministration [1], is one of the most rapidly growing areas of health. According to a global survey of the World Health Organization (WHO) there is a significant demand for the provision of generic tools to support the clinical (elearning tools, access to digital libraries, databases to support evidence-based medicine, telemedical systems, remote diagnostic systems, electronic patient records, decision support systems, etc.) and administrative (e.g., financial support systems, patient referral systems, etc.) functions of health care services in WHO member states [2]. The above is in line with the latest attempts, which have shown that the use of ICT for the provision of healthcare services across geographic, temporal, social, and cultural barriers gave a great promise to help people in isolated and remote regions to gain access in health care services [3]. 

The evolution on ehealth systems supported by advanced algorithms, high performance computers, sensors, monitoring devices, and telecommunication networks permits the provision of good quality of medical care even to remote/isolated regions, the information exchange, and the distribution of medical knowledge among health care professionals [3].

Ehealth applications have been successfully used for the provision of emergency ehealth in understaffed areas like, among others, rural health centers [4, 5] as well as for home monitoring and home care [6, 7], whenever is needed. Recent studies have shown that the home monitoring of elderly patients, as well as patients with chronic diseases, increases the individual's comfort, enhances quality of life, and encourages the patient empowerment while it reduces the number of needless transfers at hospitals and the cost of provided health care services [7–9].

Furthermore several projects deal with elderly chronic disease management as CONFIDENCE [10], ATTENTIANE [11], ENABLE [12], and K4Care [13]. 

This paper presents the architecture of the Tile-Ippokratis ehealth platform. The platform covers a wide range of services:

- support the collaboration and consultation between remote located health care personnel,
- enable the electronic monitoring of “at risk” citizens, elderly, and patients with chronic diseases, in the area of Island of Chios,
- home telemonitoring of post-surgery patients who had been provided intensive care medicine in the Intensive Care Unit of the Nicosia General Hospital; these patients were having Home Ventilation Care within their residents.

The main objectives of the project are considering:

- the collaboration between doctors and citizens located in Greece and Cyprus with different health care systems, different technology infrastructure, and different needs, in order to enhance the quality of health care services in the region South Mediterranean,
- the improvement of the self-management skills of those patients with minimum ICT skills (the ehealth platform and their electronic personal record could be accessed by them through a web browser),
- the efficient monitoring and health management of chronic and post-surgery patients by their doctors and the early response to emergency incidents using low-cost state-of-the-art web-based technology,
- the evaluation of the usability and acceptability of the proposed ehealth services in the pilot areas.

**Figure 1:**
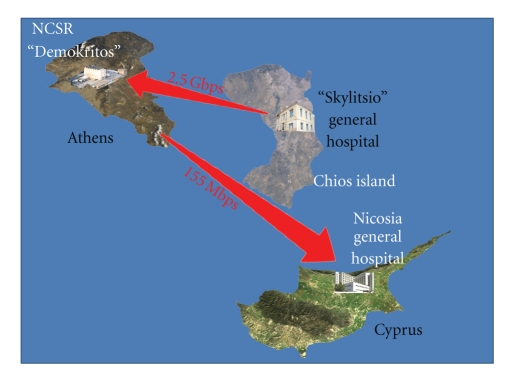
Tile-Ippokratis pilot sites.

The platform, which had been developed during the Interreg IIIA “Tile-Ippokratis” project: An INTEgrated pilot ehealth-platform for enhancing health care provision between Greece and Cyprus, has been tested and validated in the pilot sites in Greece and Cyprus (Figure 1). The platform and the provided services have been assessed by patients and physicians in the aforesaid sites. 

The platform (Server Software, EPR, Web Viewer-enabling Internet access to patient measurements) has been installed within the premises of the National Center for Scientific Research, “Demokritos”. 

In the Island of Chios the platform has been used by the Skylitsio General Hospital of Chios and the rural medical peripheral health centers residing in Chios Prefecture Municipalities of Volysos, Kardamyla, Oinouses and Pyrgi supporting elderly citizens and patients with chronic diseases. The patients were receiving support in the rural health centres.

In Cyprus the platform has been used by the Intensive Care Unit of the Nicosia General Hospital supporting post-surgery patients in the municipalities of Nicosia, Larnaca and Limassol. The patients were receiving support in their own homes.

A summary of the various services provided per pilot site and transmitted data is presented in Table 1. 

The paper describes the Tile-Ippokratis architecture, the design of the entire platform, the provided services, the used systems, and the platform infrastructure (Section 2) and presents the protocol of the trials (Section 3). In Section 4, the evaluation results of the validation phase are discussed while the conclusions are presented in Section 5.

## 2. Platform Description

The description of the platform is divided into three parts. The first part describes the teleconsultation systems and the patients' peripheral systems that entail the acquisition of the patients' biosignals and their transmission to the central database server, the second part describes the remote centralized database itself, and the third part describes the network technology selected for the uploading of the data to the database. 

### 2.1. Teleconsultation Systems and Patients' Peripheral Systems

The Tile-Ippokratis platform provides a series of services, which have been supplied through the combined use of already evaluated systems (off the self-systems as well as systems coming out of EU-funded projects) as presented in the following paragraphs.

**(1) Teleconsultation Systems**

The teleconsultation services have been mainly provided by the Wavelet-based INteractive Video Communication System (WinVicos). WinVicos is a high-end, interactive video conference system providing real-time video, still images, and audio transmission [14]. The system has been designed mainly for medical applications, like intraoperative teleconsultation, in various EU-funded projects. WinVicos supports communications via satellite, Local Area Network (LAN), Internet, ATM, xDSL, and so forth.

The teleconferencing scenario was realised with the use of a web camera connected to the computer, a set of speakers, and a microphone for the audio signal and the WinVicos software. In some cases commercially available teleconferencing software such as adobe connect (http://www.adobe.com/products/acrobatconnectpro/) has been adopted in those cases where the patients had to have routine teleconferencing sessions with their carers (Cyprus pilot). A frame rate of at least 25 fps at a resolution of 640 × 480 pixels was a preferred one for the real-time teleconferencing sessions.

Teleconferencing has been introduced for two reasons: to improve the patients' confidence to the treatment creating a feeling of safety for the patient and to support the collaboration and consultation between remote located health care personnel.

**(2) Patients' Peripheral Systems**

**Figure 2:**
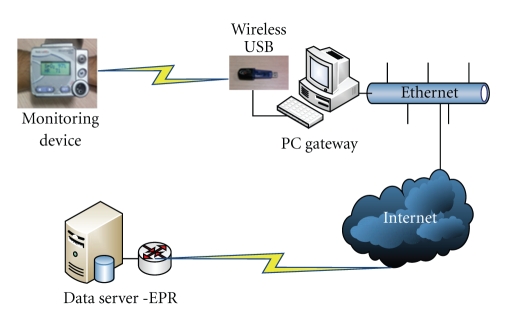
The system operation process.

For the ehealth services the following low-cost systems have been used for the provision of telemedical services in Chios Island and Cyprus. It consists of the following components:

- personal computer (PC) or laptop,
- biosignal acquisition module (Wrist ClinicTM (http://www.telcomed.ie/allinone.html)) which is responsible for biosignal collection (Figure 2); more specifically, this lightweight and mobile module is able to collect, record, store, and wirelessly transmit 1 lead ECG, SpO2, HR, BP, and TEMP up to 250 m; the device can store data if there is no connection,
- fully wireless weight scale (http://www.telcomed.ie/scale.html) which allows untethered operation (no communication or power cords); the patient can use the weight scale anywhere within coverage area (250 m communication range in open space),
- software (client) for communication with the central database (Cyprus pilot),
- application (client) for medical monitoring in public environments allowing multiple patients to use a single device (Chios pilot),
- two-way radio USB-based communication device (automatic data accumulation and transmission) which connects via ordinary internet connection; the device has 200 m communication range (open space), uses reliable data communication protocol, and has a unique digital ID for system identification. The data are transmitted from the monitoring devices to the home computer using a wireless connection (WiFi).

There is wireless connection between USB device (onboard the PC), the biosignal acquisition module, and the wireless weight scale. The selected data are stored to the PC, while an asymmetric digital subscriber line (ADSL) internet line is used for uploading data from the PC or laptop to the data base.

### 2.2. Remote Centralized Database

The EPR has been stored in an SQL-type database. The EPR information included patient details, medical info (diseases, medication), and the medical data (vital signs) acquired from the monitoring devices deployed. The data were structured in an efficient way. The medical and care personnel were able to utilize a standard web browser to login on a webpage to view a patient's record and search for readings that fall within a given range or from a specific time and date. This ability assisted medical personnel to reassure themselves about patient's condition providing the patients with an extra feeling of safety.

### 2.3. Network Description

The broadband telecommunication network integrated two types of networks, terrestrial and wireless. The network has been based on nodes of research institutes (National Center for Scientific Research “Demokritos”—NCSR), hospitals (Nikosia General Hospital and Chios Skylitsio Hospital), and the four rural peripheral medical centers in Island of Chios. Each node is connected to its appropriate router via an Ethernet link running TCP/IP protocol. The network provided broadband coverage to all the partners in Greece and Cyprus thus ensuring communication between different and remote parties allowing for data transmission and bidirectional communication by means of audio-visual content transmission. 

Through the platform clinical data have been exchanged which were collected by distributed nodes (e.g., regions supported by low-bandwidth earth network, such as rural areas in Chios Island). The uploading of the measurements data from the patients' premises to the database have been achieved either by wireless technologies—General Packet Radio Service (GPRS) data cards for PC have been used or wired technologies—Asymmetric Digital Subscriber Line (ADSL) depending on the availability of ADSL connection. ADSL was selected to be the primary technology for use in the patients' premises as well as in the Peripheral Health Centres due to the low overall cost of usage compared to the other technologies available and the provision of high speeds which can support the teleconferencing scenario as required in the project. Depending on the availability of ADSL lines, the patients' Peripheral Health Centres were connected with speeds of 1.024 Mbps download and 512 kbps upload which were sufficient for monitoring and teleconferencing. In the case of the patient's premises, these were connected with speeds of at least 512 kbps download and 128 kbps upload which were sufficient for monitoring. 

Security issues in health care information systems are of great importance. Our major objective was to allow doctors quickly access patient data when and where they needed it without compromising security. In the proposed integrated ehealth platform security, mechanisms are incorporated in order to protect the information during the transmission and storage as well as at the system level (logical access control, legitimate, and availability). For each network type an efficient security policy has been deployed, in order to protect data from unauthorized use or alteration/damage and legal issues that concern the exchange of medical data between the two countries.

The platform maintained the security of the data throughout all the communication stages, data storage, and user access to the data. More specifically the following security actions have been adopted:

- access control/single sign-on,
- authentication,
- authorization—user Access rights,
- session control,
- activity log—audit,
- data protection,
- encryption—HTTPS SSL,
- network firewalls,
- application security—application Firewall.

## 3. Protocol for the Trials

During the six-month trial phase, general practitioners and specialized doctors supported the chronic and postsurgery patients from distance, minimizing routine transportation of patients from and to hospitals. The recruitment process was based on willingness, ability, and commitment of the patients referenced by medical personnel in charge. A training process was conducted concerning the use of devices and the protocol of the monitoring program. Regular followup with users by the project partners involved scheduled reassessment, reassurance of the case management program compliance, corrective actions, reinforcement of the pilot patients and medical personnel.

Particular attention has been paid to ethical issues.

- The respect of their privacy: an internal Consortium Agreement regulated all the operations involving them with basic rules imposing the respect of their habits, their environment, and their times.
- The respect of an ethic code, ensuring a correct behavior and the respect of the patients' rhythms and attitudes. Signs of discomfort or uneasiness on the user's part during the evaluation activities have been taken into consideration.
- The collaboration with their family members: people surrounding the end users have been involved in the evaluation process. The patients have been provided with information about the purpose of the research activities, duration of their participation, and which procedures are followed.

Sixteen patients suffering from chronic diseases participated in the trials in Chios Island:

- four hypertensive patients were monitoring their HR and BP,
- four individuals with chronic pulmonary disease (chronic asthma or chronic obstructive pulmonary disease) were monitoring their SpO2 and ECG daily,
- eight individuals with chronic cardiovascular disease were monitoring their ECG, BP, and weight.

According to the rural health center scenario (Island of Chios) and the protocols that have been applied by the four General practitioners of the rural health centers together with two specialized doctors in Skylitsio General Hospital, the selected patients were measuring their vital signs frequently (2-3 times per week) in the rural health centers. Although the sixteen patients were using only four sets of devices, the vital signs through a software application for medical monitoring in public environments were automatically transmitted and saved to their electronic personal records maintained by NCSR “Demokritos”. These data were accessed frequently, at least once per week, by the medical personnel of the Skylitsio General Hospital of Chios, who were able to provide remote consultations. Based on the monitoring data and on the direct sight of the patient through the videoconference facilities the doctors from the Skylitsio General Hospital of Chios provided a second opinion to the local doctors of the rural health centers when needed.

Six postsurgery patients in Cyprus participated in the trials in Cyprus (home care trial) conducting daily from their home a full set of measurements: HR, SpO2, BP, 1 lead ECG, Temp, and weight where possible. In this scenario each patient owned one set of ehealth systems. The vital signs through the home gateway were automatically transferred and saved to their electronic personal records maintained by NCSR “Demokritos”. The medical personnel of the intensive care unit (two specialized doctors) of the Nikosia General Hospital accessed at least once per week the Electronic Patient Records providing remote consultations where necessary. The patients were able to contact their doctors via video/audio to seek medical advice. Furthermore an admin support and medical help desk has been integrated into the service concept in Cyprus pilot to motivate the patients to adhere the planned protocol. 

For the home care scenario, emergency events at home have been planned as possible to happen. The scenario concerned a patient who collects vital measurements whenever he/she does not feel well. In case abnormal situations occur and the measurements exceed set levels for a given patient, alarms triggered and doctors, family members, and emergency services automatically were notified. It has to be mentioned that during the pilot phase this scenario has been rarely conducted.

## 4. Platform Evaluation and Discussion

The evaluation of the Tile-Ippokratis ehealth platform has been primarily technical in order to verify that the systems can operate in reliable way and end users' oriented (using questionnaires and interviews) in order to investigate how the system can be embedded in everyday clinical practice. During the trials the users from both server and client sites were asked to evaluate the system, in order to evaluate not only the one-way use of the system but also capture the impressions of ICT-mediated interaction. 

Taking into account the inherent variety in the project, Tile-Ippokratis consortium opted for an evaluation approach which collects data from a variety of sources in order to ensure the integrity and generalisability of the results. To this end, data were collected through the following methods:

- Technical metrics deriving from monitoring the ehealth system during the pilots,
- Users' acceptance metrics collecting quantitative and qualitative data pertaining to the usability of the system and its users' overall experience.

### 4.1. Technical Evaluation

Tile-Ippokratis was evaluated on a number of critical technical factors which were identified as important by desk research and discussions with computer engineers and physicians. These factors guided the collection of data showing the performance of Tile-Ippokratis during its operation in the various scenarios tested during the trials; the overall results from these tests are shown in Table 2.

More specifically the first critical factor is connectivity which is defined as the capability of the ehealth system to transmit data between client and server units without any disruptions. This is the basis for the operation of any ehealth system which aims at providing continuous support to its users. Tile-Ippokratis scored high in this area having low failure rate less than 1% in the “home care” and “rural health centre” scenarios.

Ease of data transmission was the second evaluation factor. It accounts for both the ease of data transmission of biosignals and all other necessary data (e.g., video) for effective diagnosis over the network as well as the ease of data entry by users into the Tile-Ippokratis system. Given the trial results in the above-mentioned technical factor (connectivity) the outcome of ease of data transmission follows the same pattern. In the “home care” and “rural health centre” scenarios there were no problems with ease of data transmission. Data were transmitted successfully during each repetition of the trials.

The third factor quality of data transmission examines a parameter of paramount importance in telemedicine as suggested by relevant literature [15]. Data transmitted through ehealth equipment must maintain its integrity during transmission for the receiver (usually a doctor or medical personnel) to be able to produce an informed and valid opinion on the patient's condition. In both “home care” and “rural health center” scenarios, which were examined during the same trial, there were two major types of medical data transmitted through the applied network.

- Numerical data which are essentially the biosignals recorded by the system (weight, 1 lead ECG, BP, HR, SpO_2_, and temperature).
- Video-conferencing files. These were recordings automatically captured by the video-conferencing software which could be stored for subsequent review.

According to the data recorded by the project servers, in both these cases there has been no problem with the quality of data transmitted. Data was of excellent quality, and there were no complaints from both client and server units. Given the stable connectivity among the client and server sites there were no failures in the transmission of data. 

The fourth factor was interference to other medical equipments. There were no recorded problems regarding the interference of the equipment with other medical devices mainly in the rural health centers scenario. Users from both server and client units expressed a positive opinion on the interface and data entering procedures of the devices used by Tile-Ippokratis.

### 4.2. Evaluation of User Acceptance of the Ehealth Platform

Following the technical evaluation, a small-scale evaluation of the users' acceptance of the systems was performed. Whitten et al. [16] point out that users see the ehealth systems primarily as services provided to them and not as new technologies. Having this in mind and the fact that the success of every system relies on the user acceptance and user opinion must be taken into serious account as well. The evaluation of the users' acceptance of the systems included collection of data on perceived benefits for the services provided. Users were asked to fill in questionnaires regarding their experience with the interaction with the system. Interviewing them allowed us to clarify hidden attitudes and beliefs. Their views, from both server and client perspective, are summarised in Table 3.

The proposed platform was surprisingly welcomed by patients and carers because of its simplicity and functionality although most of the patients were not comfortable with using video communication and pc technology (especially the elderly one). The whole service has been viewed as an early warning mechanism which helps prevent frequent hospital visits. But although the regular user's followup and the scheduled reassessment and reinforcement of the pilot patients and medical personnel were taking place and the service was free of use for the duration of the validation phase (the project covered the communication cost together with the cost of the devices), it was observed a drop in usage rates although this was not the case according to the specified protocol. More specifically the usage rates as registered in the data base revealed that almost for the first month the users conformed to the specified medical protocol and their measurements were taken frequently as asked. But after that period the number of measurements started to gradually reduce. This behavior, mainly coming from the postsurgery patients, makes us conclude that the users are not willing to use frequently/daily and for long time periods ehealth monitoring devices even if they are asked to do it. According to Papadopoulos [17] this attitude can be explained by the fact that the patients do not perceive immediately the benefits of the mobile health applications since these services provide preventive medicine rather than cure. 

Finally, evaluation showed that Tile-Ippokratis services are reliable, from a technical perspective. No technical problems were recorded. Users considered their interaction with the system as a positive experience which makes them consider adoption of such a system in the future. Ofcourse making this platform sustainable is a real challenge especially in low-resource settings such as the Greek context. Issues such as facilitation and maintenance of communication infrastructure, payment of license fees, communication costs, and others are considered to be the problem for sustainability of such services. Maybe local authorities could undertake the responsibility to exploit and disseminate the long-term potentiality of Tile-Ippokratis services.

## 5. Conclusion

The concept and realization of integrated low cost and ease of use ehealth platform for the provision of ehealth services for both patients with chronic diseases and patients who have been served by an intensive care unit have been presented in this paper. The platform based on the use of terrestrial and wireless communication infrastructures supported teleconsultation and ehealth service. The platform ensured bidirectional communication between specialized medical personnel and civilians. The platform provided equal opportunities to all citizens to have heath care services and familiarize them more with information technology. 

The overall architecture of the platform has been technically tested while well-prepared scenarios have been evaluated. The evaluation results showed the feasibility of the proposed platform to be successfully used by physicians and civilians. Encouraged by these results the optimization of the platform for a wider delivery of telemedical services in the near future is under consideration. It is expected that the use of such platform will effectively improve the provision of health care services, reduce the related costs in terms of time and unnecessary transfers, and increasingly enhance the quality of life of individuals living in remote/isolated regions.

The results proved the functionality and utilization of the platform in Greece and Cyprus and the positive impact on psychological health of the participants. However, further actions are needed in order to enable the local health care systems and the different group population to be familiarized and use in their everyday live mature technological solutions for the provision of health care services.

## Figures and Tables

**Table 1:** Services provided per pilot site.

Connected end-points	Services	Data transferred between end-points
NCSR (server)	EPR, Web services	Incident data: personal info, contacts, medical info (diseases, medication), medical data (vital signs)

Skylitsio General Hospital (monitoring station)	Teleconsultation, ehealth	Medical data (vital signs), images

Rural medical peripheral health centers residing in Chios Prefecture Municipalities of Volysos, Kardamyla, Oinousses, and Pyrgi “at risk” citizen (client)	Teleconsultation, ehealth	Incident data: personal info, contacts, medical info (diseases, medication), medical data (vital signs), real-time video conference

Intensive Care Unit of the Nicosia General Hospital (monitoring station) postsurgery patients in the municipalities of Nicosia, Larnaca, and Limassol (client)	Teleconsultation, ehealth	Incident data: personal info, contacts, medical info (diseases, medication), medical data (vital signs), real-time video conference

**Table 2:** Overview of technical metrics of Tile-Ippokratis services.

	Connectivity with networks	Ease of data transmission
Home care	Low rate of failures (~< 1%)	No problems reported
Rural health centre	Low rate of failures (~< 1%)	No problems reported

	Quality of data transmission	Interference to other medical equipment

Home care	Excellent	None
Rural health centre	Excellent	None

	User friendliness	Wrong findings

Home care	Users satisfied, amendments asked	No wrong alarms
Rural health centre	Users satisfied, amendments asked	No wrong alarms

**Table 3:** Perceived benefits of Tile-Ippokratis services.

		Type of users	
		Physicians	Patients
Scenario	Home care	Good alternative for avoiding frequent visits to the hospital Long term-appreciation of patient's situation—useful in cases of emergency Shifting of the location of care, out of hospitals	Increased sense of safety Avoidance of unnecessary visits to the hospital Increased sense of “always connected” with their doctors
	Rural health center	Sense of “team” work between remote located medical personnel Time savings from patients' transportation to Skylitsio General Hospital and other large hospitals Increased sense of safety because of the second opinion choice Low-cost ehealth services. One ehealth system for many patients Ensuring continuity of healthcare and patient followups	Larger number of patients own their electronic personal record Increased positive attitude against ICT technologies Promotion of behavioural modification, goal setting, prevention rather than treatment and adjusting to living with a chronic disease improving patients' feeling of safety Assist patients to self-manage their chronic condition and enhance supportive measures to promote self-management in the long term
